# Supplementary material for: MicroRNA-148a Regulates the Proliferation and Differentiation of Ovine Preadipocytes by Targeting PTEN
Source: Animals (Basel). 2021 Mar 15;11(3):820. doi: 10.3390/ani11030820 (PMC7998426; doi:10.3390/ani11030820)
Supplement: Supplementary file 1 [file animals-11-00820-s001.pdf]

Gene	Forward primer sequence (5'–3')	Reverse primer sequence (5'–3')	Reference sequence
aP2	GTCCTTCAAATTGGGCCAGG	ACTCTGGTAGCAGTGACACC	NM_001114667.1
PPARγ	GAGCCTTCCAACTCCCTCAT	ATGAGACATCCCCACAGCA A	NM_001100921.1
C/EBPβ	CATCGACTTCAGCCCCTACC	CCGCCTTCTTGCAGTTCTTG	XM_004014883.4
FATP4	CTGTCCTTCGTGTACCCCAT	CTTCTTGTTGTTGGCGCTCT	XM_015094163.2
LPL	AGCTGCAGAAAGAACCGTTG	GAGATCTCGAAGGCCTGGTT	NM_001009394.1
FASN	ACACAAATTGAGCAGCCCTG	TGCCGCTCTTGTACACTGTA	XM_027974304.1
GLUT4	TGGCTACAACATTGGGGTCA	ATCATGCCACCCACAGAGA A	XM_027974995.1
PTEN	AAGCTGGAAAGGGACGAACT	ACACATAGCGCCTCTGACTG	XM_027960248.1
CDK2	GACCAGCTCTTCCGGATCTT	ACAAGCTCCGTCCATCTTCA	NM_001142509.1
CDK4	ACTTTGTGGCCCTCAAGAGT	CCTGAGGTCTTGGTCCACAT	NM_001127269.1
cyclin B1	CCCTCCAGAAATCGGTGACT	AGCTCAACATCAACCTCTCC A	XM_027980034.1
PCNA	TCAAGTGGCGTGAACCTACA	TACTAGTGCCAAGGTGTCCG	XM_004014340.4
P53	CGGCTTGCAGAAACCTCTTT	CCCTTTTCTACCTCCTGCCA	XM_004006850.4
TBP[1]	ACAGCCTCCCACCATATGCCC	GCTGTGGAGTCAGTCCTGTG C	XM_027972700.1
U6[1]	TGGAACGCTTCACGAATTTGCG	GGAACGATACAGAGAAGAT TA	XM_012096728.2
PTEN 3'UTR-wild[2]	CCGCTCGAGAATACAGATTGC GTAGGACC	AAATATGCGGCCGCGAAAG ATGTTCAAGAGGAGC	XM_027960248.1
PTEN 3'UTR-Mut[3]	GGGCTTTACGTGACTTATTATTT TTCCTTTGGAATGTGAAG	TAAGTCACGTAAAGCCCATT ATAATGTCATTATTTATGA	XM_027960248.1
PTEN CDS[4]	GGGGTACCGCCACCATGACAG CCATCATCAAAGAGATC	GCTCTAGATCAGACTTTTGT AATTTGTGTATGCTG	XM_027960248.1

Table 1. List of the primers used in this study

[1] *TBP* and *U6* were chosen as internal reference genes as suggested by Birsoy et al. (2008) and Zhang et al. (2018).

[2] Primers are used to amplify the wild-type 3'UTR sequence of the target gene

[3] Primers are used to amplify the mutant-type 3'UTR sequence of the target gene

[4] Primers are used to amplify the coding sequence of the target gene
